# Dressing Impregnated with Chlorhexidine and Vancomycin for the Prophylaxis of Central Venous Catheter-Related Infections—A Randomized Trial

**DOI:** 10.3390/idr17040102

**Published:** 2025-08-19

**Authors:** Giovanna Cerri Lessa, Carolina Comitti Zanella, Gustavo Pessatto Krause, Alexandre Moreira Senter, Paula Hansen Suss, Gabriel Burato Ortis, Thyago Proenca de Moraes, Felipe Francisco Tuon

**Affiliations:** Laboratory of Emerging Infectious Diseases, School of Medicine, Pontifícia Universidade Católica do Paraná, Curitiba 80215-901, Brazil; giovannacerri@hotmail.com (G.C.L.); carolcomitti@hotmail.com (C.C.Z.); gustavokrause@hotmail.com.br (G.P.K.); ale.senter@gmail.com (A.M.S.); paula.h@pucpr.br (P.H.S.); gabrielburatortis@hotmail.com (G.B.O.); thyago.moraes@pucpr.br (T.P.d.M.)

**Keywords:** dressing, vancomycin, chorhexidine

## Abstract

**Background:** Central venous catheters (CVCs) are essential in intensive care units (ICUs) for monitoring and administering treatments; however, catheter-related bloodstream infections (CRBSIs) are significant complications, leading to severe outcomes and increased healthcare costs. The objective of this study was to evaluate the effectiveness of a simple and inexpensive impregnated dressing (intervention) compared to a non-impregnated dressing in reducing catheter-related infections among critically ill patients using vancomycin and chlorhexidine. **Methods**: This was a randomized, double-blind, controlled clinical trial in a university hospital in Brazil with 207 beds from June 2022 to October 2023. Patients over 18 years old admitted to the ICU and needing a CVC for a period exceeding 72 h were included. A CVC inserted outside the ICU and the need for two CVCs in the same patient simultaneously were exclusion criteria. One group received an impregnated dressing (intervention) compared to the other group, which received a standard dressing (comparator). The incidence of CRBSIs and the microbiological outcomes were evaluated. The primary endpoint was CRBSI. **Results**: The clinical trial included 516 patients randomized to receive either the new antimicrobial dressing or a control dressing. The dressing significantly reduced CVC colonization but not CRBSI rates. **Conclusions**: This new dressing provides enhanced antimicrobial protection but does not decrease CRBSI incidence. Future studies should further explore the cost-effectiveness and long-term benefits of this approach.

## 1. Introduction

The use of central venous catheters (CVCs) in critically ill patients in intensive care units (ICUs) is nearly routine due to the necessity for specific monitoring, as well as the administration of vasoactive drugs that cannot be delivered through peripheral venous access, allowing for rapid rehydration, the administration of vasoactive drugs, hemodynamic monitoring, and parenteral nutritional support, among other functions [1,2]. However, catheter-related bloodstream infection (CRBSI) is one of the most concerning adverse events, leading to the development of sepsis and its complications, including endocarditis, septic embolism, and even death, in addition to increasing antimicrobial consumption and imposing costs on the healthcare system [3]. The rate of CRBSI varies significantly among hospitals in developed countries, ranging from 0.1 to 1 per 1000 CVC days [4,5].

Currently, many CVC care bundles are being implemented to prevent CRBSIs, including hand hygiene, maximal barrier precautions during insertion, and skin antisepsis, among others [6]. In recent years, the use of chlorhexidine for CRBSI prevention has garnered attention from healthcare providers. Numerous studies have reported on chlorhexidine applications in various forms, such as chlorhexidine bathing, skin disinfection, oral hygiene, and impregnated dressings [7]. Based on a literature review, several randomized controlled trials (RCTs) have assessed the role of chlorhexidine-impregnated dressings in prophylaxis against CVC-related complications. Three meta-analyses have already shown that chlorhexidine-impregnated dressings for prophylaxis against CVC-related complications are beneficial [8,9,10]. However, this type of dressing is not routinely used in hospitals due to the high costs for public hospitals and the private/complementary healthcare system; the reimbursement rates from health insurers do not cover these expenses.

Another issue that has arisen in recent years is the increasing resistance of *Staphylococcus aureus* and coagulase-negative *Staphylococcus* to chlorhexidine, leading to the potential ineffectiveness of these dressings according to local epidemiology, which can exceed 30% in methicillin-resistant *S. aureus* [11,12,13,14]. Considering that there has been a progressive increase over the years for chlorhexidine, its combination with vancomycin could increase coverage against the main pathogens of CRBSI, which are Gram-positive cocci. The combination of vancomycin with a dressing may pose a risk for the development of resistance; however, cases of vancomycin-resistant *S. aureus* are rare. Furthermore, vancomycin consumption in hospitals is high, making it one of the most widely used antibiotics, which promotes the selection of multidrug-resistant bacteria in various microbiotas, such as the intestinal microbiota, which is populated by *Enterococcus*. It is speculated that the risk of resistance from its localized use on the skin should not be considered a major concern. Another issue that should be considered when using vancomycin in a dressing is its long-term stability, which allows for an extended shelf life.

Taking into account resistance to chlorhexidine as a potential risk for the failure of dressings impregnated with this antimicrobial, we developed a dressing impregnated using a combination of vancomycin with chlorhexidine and evaluated its impact on CRBSI rates.

## 2. Materials and Methods

### 2.1. Pre-Clinical Study—Dressing Validation

For the development of the product, cellulose in the form of filter paper with a weight of 150 g was used. Disks with a 20 mm diameter were made with a central hole of 5 mm for the passage of the catheter. The paper was immersed in a solution of 4% germicidal chlorhexidine with vancomycin 25 mg/mL for 10 min. The dressing was then subjected to freezing at −80 °C and then subjected to lyophilization and sterilized with ethylene oxide.

The skin allergy test showed no allergic reactions in any of the volunteers up to day 14. The dressings removed from these donors were placed in culture plates, and no pathogen growth was observed. When these dressings were placed on plates seeded with *S. aureus* ATCC 25923, antimicrobial activity was maintained.

### 2.2. Study Setting

This is a randomized, double-blind, placebo-controlled clinical trial evaluating the incidence of CRBSIs in ICU patients using antimicrobial-impregnated CVC dressings. The analysis period spanned from June 2022 to October 2023. The study was conducted at a renowned center for trauma and surgery localized in Southern Brazil, with a total of 207 beds, including 29 beds in the general ICU. The rate of CRBSI was 2.4 per 1000 CVC days (between 2017 and 2021). This study was approved by a local ethical committee (Pontificai Universidade Catolica do Parana, number 43295821.5.0000.0020, February, 02, 2021, entitled “Curativo impregnado com clorexidina e vancomicina para a profilaxia de infecções relacionadas a cateter venoso central”).

### 2.3. Study Design

This was a randomized, double-blind, controlled clinical trial for patients undergoing CVC use in the ICU. The Consolidated Standards of Reporting Trials (CONSORT) checklist was used for this paper (Supplementary Material S1—Table S1). A pre-clinical study was designed to validate the spectrum and antimicrobial activity (Supplementary Material S1—Table S1). Every patient indicated for a CVC was randomized after signing the informed consent form, or after a responsible family member had signed it on their behalf. Randomization was conducted by the lead intensivist (non-blinded).

### 2.4. Eligibility Criteria

Inclusion criteria included being over 18 years old, being admitted to the ICU, needing a CVC inserted in the ICU by a trained team, using the CVC for a period exceeding 72 h, and having intact skin in the area of contact with the dressing. Having the CVC inserted outside of the ICU and the same patient needing two CVCs simultaneously were exclusion criteria. This is because the subsequent CVC procedure could not be randomized. Other infections were not excluded during the evaluation. Only short-term CVCs were evaluated (long-term CVCs and hemodialysis were excluded).

### 2.5. Interventions

The dressing was produced in the Laboratory of Studies and Innovation in Medical Devices (LEID) using a combination of 4% chlorhexidine and vancomycin (25 mg/mL) and was sterilized with ethylene oxide. The central venous catheter insertion procedure followed the Seldinger technique. The nurse responsible for applying the dressing after catheter placement and the patient were blinded to the treatment group. The control group received the same dressing material without impregnation.

Following the determination of the puncture site—either in the internal jugular vein, subclavian vein, or femoral vein—skin antisepsis was performed using alcoholic chlorhexidine, followed by the placement of sterile drapes. Local anesthesia was then administered, followed by venous puncture according to the anatomical landmarks of each site. Once the vein was punctured, a guidewire was inserted, and the needle was removed. After this step, a small dilation of the skin and subcutaneous tissue was performed using a dilator, followed by the definitive placement of the double-lumen catheter and removal of the guidewire. The procedure was completed by performing a blood reflux test through the catheter lumens to ensure proper positioning, followed by securing the catheter to the skin with 3.0 nylon sutures.

### 2.6. Variables and Clinical Outcomes

Patients were not undergoing any other researcher interventions regarding other procedures, and the decision to maintain or remove the CVC remained at the discretion of the attending team. Epidemiological data such as sex, age, comorbidities, severity scores at admission (APACHE and SOFA), reason for ICU admission, need for mechanical ventilation, and clinical outcomes were evaluated. Data related to the CVC include insertion site, duration of CVC use, number of attempts at insertion, compliance with the bundle of measures for CVC infection prevention, and indication for CVC removal (no longer needed, malfunction, or suspected infection). The hospital CRBSI rates before and after the study were also evaluated, expressed as the monthly number of infections per 1000 CVC days. CRBSI was the primary endpoint, and the secondary outcome was mortality.

### 2.7. Microbiological Study of CVC

For microbiological tip culture of CVCs, all removed CVCs were sectioned and the distal 2 cm was subjected to freezing and stored at 80 °C in glycerol. Further, the catheter was cultured using the rolling technique, with quantitative and semi-quantitative culture [15]. Only clinically suspected cases of infection were referred to the local laboratory for CRBSI diagnosis, as described below.

### 2.8. CRBSI Definition

CRBSI was defined according to the criteria of the IDSA, which include the presence of bacteria compatible with CRBSI in blood culture, clinical signs of systemic infection, absence of another focus, and the identification of the same pathogen from blood culture in the catheter tip culture [16]. CRBSI was the primary endpoint of the study.

### 2.9. Sample Size

The sample size was defined as at least 300 patients in each group, based on a 50% reduction in CRBSI with the use of the impregnated dressing, a current incidence of 2.4 cases/1000/CVC/day, and a standard deviation of 5 over the last 18 months. A sample power of 80% and *p* < 0.05 were used.

### 2.10. Statistical Analysis

Our null hypothesis was that a dressing impregnated with chlorhexidine and antibiotics would have no effect on the CRBSI rate in a cohort of patients with CVCs. In contrast, the alternative hypothesis posited that an impregnated dressing would reduce the rate of catheter-related infections. Categorical data were described as percentages and continuous data were described as means or medians according to the distribution pattern (normality). The standard deviation (SD) and 25% and 75% interquartile ranges (IQRs) were used as distribution variables for the mean and median, respectively. Risk factors associated with outcomes (CRBSI or insertion site infection) were calculated according to each variable and its distribution, as determined by Student’s *t*-test, a Mann–Whitney test, a chi-square test, or Fisher’s exact test. A difference of less than 5% (*p* < 0.05) was considered statistically significant. For multivariate analysis, all variables with statistical significance in the univariate analysis were included in a logistic regression to be defined. Adjustment for severity and mechanical ventilation was included and is detailed in the Supplementary Materials—Supplementary Figure S1 and Supplementary Table S2.

## 3. Results

### 3.1. Participants and Recruitment

**Figure 1:**
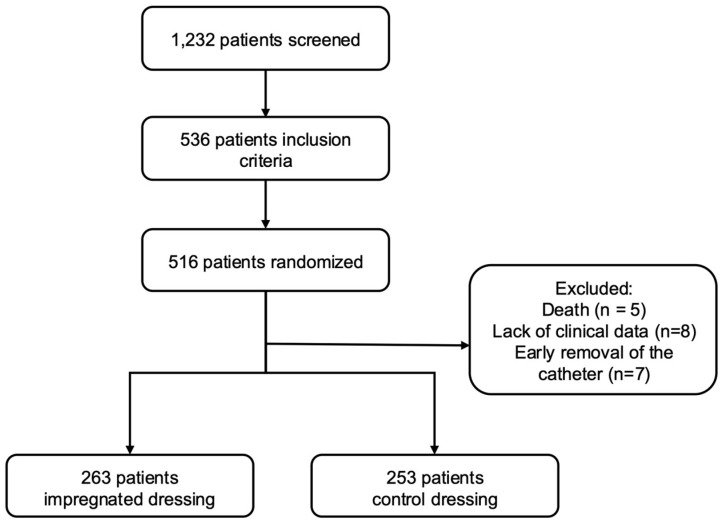
Flowchart of patients with central venous catheter included in this study of an impregnated dressing.

A total of 1232 patients were screened, but only 536 met the inclusion criteria during the enrollment period. Of these, 20 were excluded after randomization due to early death (<72 h of CVC) (*n* = 5), lack of clinical data (*n* = 8), and early removal of the catheter (*n* = 7). Of the 516 randomized patients, 263 were included in the impregnated group and 253 in the control group (Figure 1).

### 3.2. Baseline Data and Number Analyzed

The median age was 60 years [IQR 43.2–70], with 35.4% being female. The median hospital length of stay was 23 days [IQR 13–42], the median ICU stay was 11 days [IQR 6–20], the SOFA score was 8 [IQR 6–10], and the SAPS score was 62 [IQR 49–75]. The overall mortality rate was 44.5% (*n* = 230), and 90.5% (*n* = 467) were on mechanical ventilation at some point during their hospital stay (Table 1).

The reasons for hospital and ICU admission were divided into nine categories: polytrauma with traumatic brain injury (TBI), polytrauma without TBI, cardiovascular causes, non-traumatic neurological causes, infectious causes, renal or metabolic causes, elective postoperative reasons, gastrointestinal causes, and pulmonary causes. Among the reasons for hospital admission, 18.6% of patients were admitted for polytrauma with TBI, 13% for polytrauma without TBI, 23.2% for cardiovascular causes, 17.6% for non-traumatic neurological causes, 12.6% for infectious causes, 1.4% for renal or metabolic causes, 1.4% for elective postoperative reasons, 9.7% for gastrointestinal causes, and 2.3% for pulmonary causes. The reasons for ICU admission did not always match the reasons for hospital admission, with 22.8% admitted to the ICU for polytrauma with TBI, 9.1% for polytrauma without TBI, 21.7% for cardiovascular causes, 16.6% for non-traumatic neurological causes, 12% for infections, 1.4% for renal or metabolic causes, 3.1% for elective postoperative reasons, 10.8% for gastrointestinal causes, and 2.5% for pulmonary causes.

Of the 516 patients in the study, 230 (44.6%) passed away, either in the ICU or during hospitalization. The Charlson Comorbidity Index was 0 in 275 patients (53.3%), indicating no significant comorbidities. However, 40.8% of patients had a Charlson Comorbidity Index between 1 and 3, and 6.1% had an index between 4 and 8. The Charlson Comorbidity Index in the control group was 0 in 143 patients (56.5%), indicating no significant comorbidities; 39.1% of patients had a Charlson Comorbidity Index between 1 and 3, and 4.4% had an index between 4 and 7. The Charlson Comorbidity Index in the impregnated group was 0 in 132 patients (50.2%); 42.2% of patients had a Charlson Comorbidity Index between 1 and 3, and 7.6% had an index between 4 and 8.

Overall, 9.5% of participants did not require invasive mechanical ventilation during their stay, while 90.5% needed it. Furthermore, 54% of the study patients experienced acute or exacerbated chronic renal dysfunction, including those classified as KDIGO 2 or 3 or requiring renal replacement therapy for any reason.

Indications for central venous catheter placement were divided into four categories: shock (85.9%), monitoring (10.5%), inability to access peripheral veins (2.3%), and the need for parenteral nutrition (1.4%). The catheter was inserted into the jugular vein for 61.9% of patients and in the subclavian vein for 37.7%. Of the patients, 6.2% had a central catheter-related infection.

### 3.3. Outcomes and Estimation

There were 17 cases in the control group and 15 cases in the impregnated dressing group (primary outcome). In the univariate analysis, mechanical ventilation (RR = 1.12 [1.04–1.14]; *p* = 0.037) and renal failure (RR = 2.27 [1.03–5.02]; *p* = 0.027) emerged as significant risk factors for CRBSI. None of the other categorical variables, including the type of dressing (impregnated vs. control), were identified as risk factors. The bundle compliance was similar during both periods of the study (before = 97% vs. after = 96%).

Mortality appeared to be higher in the intervention group (47.5% vs. 41.5%); this is not a negligible difference, even if it is not statistically significant. Mortality among patients with CRBSI was significantly higher (RR = 2.91 [1.35–6.29], 68.7% vs. 42.9%; *p* = 0.004). In the analysis of continuous variables, the univariate analysis identified several risk factors for CRBSI. These included length of ICU stay (mean = 26.7 ± 16.7 days vs. 14.1 ± 12.1 days; *p* < 0.001), duration of dressing use (mean = 9.7 ± 7.0 days vs. 6.9 ± 5.8 days; *p* = 0.001), and SOFA score (mean = 9.6 ± 2.8 vs. 8.0 ± 3.7; *p* = 0.014).

In the multivariate analysis, the only independent risk factors for CRBSI identified were length of ICU stay (*p* < 0.001) and duration of dressing use (*p* = 0.043). Of the 253 patients in the control group, 105 passed away, while 125 patients in the intervention group (263 in total) had the same outcome (*p* = 0.099). In the control group, 220 patients were under mechanical ventilation during hospitalization, compared to 247 in the intervention group (*p* = 0.005). In the control group, 135 patients experienced renal dysfunction, compared to 144 in the intervention group (*p* = 0.4), and 17 patients had a CRBSI, versus 15 in the intervention group (*p* = 0.384).

### 3.4. Microbiological Evaluation of Tip CVC

All removed catheters were sent for culture, with 68 cultures returning positive results: 16 in the impregnated group (16/263; 6%) and 52 in the control group (52/253; 20%). Comparison between the two groups showed a higher positivity rate in the control group compared to the impregnated group. The details of the identified pathogens are described in Table 2. Across the 68 microorganisms identified, Gram-positive bacteria were the most frequently isolated, particularly coagulase-negative staphylococci, with *Staphylococcus* epidermidis accounting for the majority of cases (*n* = 19). Notably, 17 of these isolates were from the control group, whereas only 2 were detected in the impregnated dressing group, suggesting a possible reduction in this common skin contaminant with the intervention. Gram-negative organisms were less frequently isolated, with *Klebsiella oxytoca* and *Pseudomonas aeruginosa* being the most notable species, though their distributions did not appear to differ significantly between groups. *Candida* species (*n* = 5) and polymicrobial infections (*n* = 5) were also present, again showing no clear predominance in either group. Overall, while the control group yielded a higher total number of isolates (52 vs. 16), the findings suggest that the impregnated dressing may have contributed to a lower recovery of certain Gram-positive organisms, particularly coagulase-negative staphylococci, which are commonly implicated in catheter-related bloodstream infections.

## 4. Discussion

This study did not find a significant difference in central catheter-related infections between the two groups, even though the control group had a higher rate of tip-positive cultures (20%) compared to the impregnated group (6%). This finding suggests that the impregnated dressings may be effective in reducing bioburden and catheter colonization. Theoretically it could be advantageous, with a potential reduction in CRBSIs. Nevertheless, the institutional CRBSI rate analysis showed a trend toward decreased incidence post-study, with the median rate dropping from 3.3 to 2.1 CRBSI per 1000 CVC-days. This reduction suggests that the introduction of the impregnated dressings may have contributed to a lower incidence of CRBSIs in the broader institutional setting, supporting their potential utility in infection control strategies. Another possibility represents a possible bias associated with this study, with the impregnated dressings promoting better adhesion to the CVC bundle.

In our study, CRSBI was associated with an increased risk of mortality in comparison to those without infection. CRBSIs have a significant clinical and economic impact, as they are associated with higher rates of mortality, morbidity, hospital costs, and therapeutic challenges. This impact can be higher with multidrug resistance (MDR). A previous study showed that 30-day mortality was significantly higher in patients with infections due to MDR agents than those due to non-MDR agents (52.75 versus 21.3%) [17]. In addition to increased mortality, studies show that MDR bloodstream infections result in longer hospitalizations with higher costs [18], in addition to increasing the likelihood of hospital readmissions [19]. Mortality appeared to be numerically higher in the intervention group (125 vs. 105 deaths), and this is not a negligible difference, even if it is not statistically significant. It is important to note that patients in the intervention group presented with higher baseline SAPS scores, were older, and had greater need for mechanical ventilation, which may have influenced the outcomes.

Catheter-related infections are a complication that greatly influences the morbidity and mortality of patients using CVCs, a fact that justifies unceasing efforts to promote preventive evidence-based interventions. CRBSIs are considered the most preventable nosocomial infection [20]. We found some risk factors associated with CRBSIs, which included mechanical ventilation and renal failure; however, the most important was the higher mortality associated with CRBSIs, as previously discussed.

The most common routes for CVC contamination are the migration of skin microorganisms at the catheter insertion site along its surface tract, with subsequent colonization of the catheter tip, and direct contamination of the catheter hub through contact with hands, contaminated fluid, or devices. Less commonly, catheters might be contaminated hematogenously from another focus of infection [21]. It is the pathophysiology of catheter contamination that explains why impregnated dressings are important for preventing catheter-related infections, since due to their antimicrobial effect they do not allow for bacterial growth around the catheter insertion site, inhibiting colonization. The impregnation of the dressing with vancomycin may have directly influenced the microbiological profile observed between groups. The markedly lower recovery of coagulase-negative staphylococci, particularly *Staphylococcus epidermidis*, in the impregnated group (2 isolates) compared to the control (17 isolates) suggests that the local presence of the antibiotic may have acted as a barrier against these Gram-positive pathogens, which are commonly associated with catheter-related infections. On the other hand, the occurrence of Gram-negative microorganisms, such as *Klebsiella* spp. and *Pseudomonas* spp., did not show a notable reduction, which is expected since vancomycin has no activity against these bacteria. This finding raises the hypothesis that the use of a vancomycin-impregnated dressing may create selective pressure favoring microorganisms intrinsically resistant to this antibiotic, such as Gram-negative bacilli and *Candida* spp., although the number of isolates does not allow for definitive conclusions. Overall, these data suggest the potential benefit of reducing Gram-positive contamination while also underscoring the importance of monitoring the broader microbiological impact and the potential risk of resistance selection.

The cost of this new dressing is extremely low, equivalent to USD 0.10. Obviously, the indirect costs and the need for large-scale manufacturing and testing required by regulatory agencies were not taken into account. But even in this sense, the costs would not exceed those of current commercially available dressings, whose cost–benefit analysis is very important. When you introduce a new technology at a low cost, the cost–benefit becomes much easier to evaluate, especially in low- to middle-income countries. Using a lower-cost dressing for central venous catheter care could result in substantial economic benefits. At just USD 0.10 per dressing compared to the concurrent option at USD 9.70, the savings per patient amount to USD 9.60. Applied to 1232 patients, this translates to a total saving of USD 11,827.20. Such a reduction in expenditure could free up resources for other areas of patient care, improve overall hospital cost efficiency, and make high-quality catheter management more affordable without compromising clinical standards—provided the lower-cost dressing maintains equivalent safety and effectiveness.

Large studies comparing impregnated dressings with standard dressings have demonstrated a significant reduction in the rate of bloodstream infections, which is mainly due to their antimicrobial properties. A previous meta-analysis showed that dressings impregnated with chlorhexidine significantly reduce the risk of catheter colonization (RR = 0.46, 95% CI: 0.35 to 0.58) and the incidence of catheter chain infection-related blood pressure changes (RR = 0.6, 95% CI: 0.42 0.85) [10]. Timsit et al., in a randomized clinical study, with a total of 1879 patients evaluated, demonstrated a 60% reduction in the rate of CRBSIs (RR 0.402 CI 95% 0.186–0.868, *p* = 0.02) [22]. Xu et al. (2024) performed a meta-analysis that showed that chlorhexidine-impregnated dressings, compared to standard polyurethane dressings, can reduce the incidence of BSIs (RR 0.60, 95% CI 0.44–0.83) and the colonization of the catheter tip (RR 0.7, 95% CI 0.52–0.95) [7].

Using a topical dressing containing vancomycin for skin wounds raises concern about the potential development of vancomycin-resistant organisms. When vancomycin is applied directly to the skin, local bacteria are exposed to sub-therapeutic or inconsistent drug concentrations, which can create selective pressure favoring the survival and proliferation of resistant strains, including vancomycin-resistant *Enterococci*. This risk is particularly relevant if the dressing is used over prolonged periods, on large wound surfaces, or in settings with high bacterial load. Additionally, the widespread use of topical vancomycin could contribute to a broader reservoir of resistance genes in the environment, which might later lead to more clinically significant pathogens [23].

This study has several limitations that should be acknowledged. First, although the impregnated dressing reduced catheter colonization, it did not significantly lower CRBSI incidence, suggesting that the sample size or follow-up duration may have been insufficient to detect a meaningful difference. The trial was conducted in a single center with a specific ICU patient population, which limits the generalizability of the findings. Only short-term central venous catheters were evaluated, excluding long-term or hemodialysis catheters. Although the study was randomized, the lead intensivist was not blinded, potentially introducing bias. Moreover, improved adherence to infection control protocols during the study period may have influenced the outcomes (Hawthorne effect). The prophylactic use of vancomycin in the dressing raises concerns about the development of antibiotic resistance, particularly among Gram-positive organisms such as *Enterococcus*, despite its topical application. Skin safety evaluation was limited to five healthy volunteers over 14 days, which may not adequately reflect the safety profile in critically ill patients or with long-term use. However, during the clinical trial, no case of allergy was identified. Additionally, while the dressing is low-cost, no formal cost-effectiveness analysis was conducted, which is essential for broader implementation, especially in resource-limited settings. Lastly, the study did not report detailed compliance data with the CVC care bundle, a factor that could have significantly impacted infection rates.

## 5. Conclusions

In conclusion, the impregnated dressings do not demonstrate a significant reduction in central catheter-related infections. While they did not show a significant impact on mortality, their potential benefits in reducing infection rates make them a valuable tool in critical care settings. Further research is needed to fully elucidate their role in improving patient outcomes and optimizing infection control practices.

## Figures and Tables

**Table 1:** Clinical and epidemiological variables of patients with impregnated or non-impregnated (control group) central venous catheter dressing.

		Control		Impregnated		*p* Value
Variables		(*n* = 253)		(*n* = 263)		
Gender (male)		168	66%	165	63%	0.218
Age (years)		58	[42–70]	63	[45–72]	0.049
Charlson index score [median, IQR]		0	[0,1]	0	[0–2]	0.081
SOFA score		8	[5–10]	8	[6–11]	0.197
SAPS score		58	[46–71]	63	[52–76]	0.004
Mechanical ventilation		220	87%	247	94%	0.005
Length of stay (days)		22	[12–42]	24	[13–45]	0.575
ICU length of stay		10	[6–17]	12	[6–20]	0.128
Dressing use (days)		5	[3–7]	5	[4–8]	0.456
Death		105	41.5%	125	47.5%	0.099
Catheter-related infections		17	6.7%	15	5.7%	0.384
Indication for CVC						
	Vasoactive drugs	223	88%	220	84%	
	Invasive monitoring	24	9%	30	11%	
	Parenteral nutrition	4	2%	8	3%	
	Peripheral venous catheter unavailable	2	1%	5	2%	

OFA—Sequential Organ Failure Assessment; SAPS—Simplified Acute Physiology Score; ICU—intensive care unit; CVC—central venous catheter.

**Table 2:** Microorganisms identified in central venous catheter tip removed from patients with impregnated or non-impregnated (control) dressing.

Microorganisms				Total (*n*)	Impregnated (*n*)	Control (*n*)
Gram-positive						
	Coagulase-negative					
		*Staphylococcus epidermidis*		19	2	17
		*Staphylococcus capitis*		3	1	2
		*Staphylococcus hominis*		2	0	2
		*Staphylococcus haemolyticus*		1	1	0
		*Staphylococcus* spp.		1	0	1
	*S. aureus*			3	1	2
	*Micrococcus* spp.			4	1	3
	*Enterococcus faecalis*			3	1	2
	*Corynebacterium lipophilic*			2	0	2
	*Corynebacterium* spp.			1	0	1
Gram-negative						0
	Fermenter bacteria					0
		*Klebsiella oxytoca*		5	0	5
		*Serratia marcescens*		2	0	2
		*Enterobacter cloacae complex*		1	1	0
		*Klebsiella pneumoniae*		1	0	1
	Non-fermenter bacteria					0
		*Pseudomonas putida*		1	0	1
		*Acinetobacter baumannii*		4	1	3
		*Pseudomonas aeruginosa*		5	2	3
						0
*Candida albicans*				5	2	3
Polymicrobial				5	3	2
Total				68	16	52

## Data Availability

Data are available under request.

## Supplementary Materials

The following supporting information can be downloaded at https://www.mdpi.com/article/10.3390/idr17040102/s1: Table S1: CONSORT 2010 checklist of information to include when reporting a randomised trial; Table S2: Impact of the intervention on Infection. Sensitivity analysis for tertiles of risk scores; Figure S1: Impact of the intervention on Infection. Adjusted models.

## Abbreviations

The following abbreviations are used in this manuscript:

ICU	Intensive care unit
CRBSI	Catheter-related bloodstream infection
CVC	Central venous catheter
